# Migration of an Escape Room–Style Educational Game to an Online Environment: Design Thinking Methodology

**DOI:** 10.2196/32095

**Published:** 2022-09-26

**Authors:** Maja Videnovik, Tone Vold, Georgina Dimova, Linda Vibeke Kiønig, Vladimir Trajkovik

**Affiliations:** 1 Center for Innovations and Digital Education Dig-Ed Skopje North Macedonia; 2 Department of Business Administration and Organizational Studies Inland Norway University of Applied Sciences Elverum Norway; 3 Faculty of Computer Science and Engineering Ss Cyril and Methodius University Skopje North Macedonia

**Keywords:** digital games, escape room, computational thinking, gamification, students’ engagement, interactive learning environments, serious games, digital health, education, student education, learning outcomes, digital learning, digital education, educational games

## Abstract

**Background:**

The COVID-19 pandemic outbreak has led to a sudden change in education, closing schools and shifting to online teaching, which has become an enormous challenge for teachers and students. Implementing adequate online pedagogical approaches and integrating different digital tools in the teaching process have become a priority in educational systems. Finding a way to keep students' interest and persistence in learning is an important issue that online education is facing. One possible way to establish engaging and interactive learning environments, using the energy and enthusiasm of students for educational purposes, is the use of game-based learning activities and gamification of different parts of the educational process.

**Objective:**

This paper presents a use case of migrating an escape room–style educational game to an online environment by using the design thinking methodology. We wanted to show that the design thinking methodology is useful to create engaging and motivating online games that provide educational value.

**Methods:**

Starting from students’ perspective, we created a simple digital escape room–style game where students got an opportunity to self-assess their knowledge in computer science at their own pace. Students tested this prototype game, and their opinions about the game were collected through an online survey. The test's goal was to evaluate the students' perceptions about the implemented digital escape room–style educational game and gather information about whether it could achieve students' engagement in learning computer science during online teaching.

**Results:**

In total, 117 students from sixth and seventh grades completed the survey regarding the achieved student engagement. Despite the differences in students’ answers about game complexity and puzzle difficulty, most students liked the activity (mean 4.75, SD 0.67, on a scale from 1 to 5). They enjoyed the game, and they would like to participate in this kind of activity again (mean 4.74, SD 0.68). All (n=117, 100%) students found the digital escape room–style educational game interesting for playing and learning.

**Conclusions:**

The results confirmed that digital escape room–style games could be used as an educational tool to engage students in the learning process and achieve learning outcomes. Furthermore, the design thinking methodology proved to be a useful tool in the process of adding novel educational value to the digital escape room–style game.

## Introduction

Education and teaching nowadays are shifting to a learner-centered approach and skill development where a teacher guides students in the learning process, enabling them to progress at their own pace, considering different students' learning styles. Different digital tools and learning paradigms are often included in the education in order to increase the quality of achieved learning [1].

The outbreak of COVID-19 has led to a sudden change in education. More than a billion students worldwide have been affected by school and university closures due to the COVID-19 pandemic [2,3]. The affected number of students equals around 90% of the world’s enrolled students [2,4].

This situation has led shifting to online teaching, which has become an enormous challenge for teachers and students. There has been a remarkable rise in e-learning, whereby teaching is undertaken remotely and on different digital platforms [5]. Teachers have been facing a big challenge in how to adapt teaching materials and methodologies to an online environment. Achievement of educational goals, planning online activities, keeping students' interest and motivation, and assessing gathered knowledge and skills are just some of the issues that teachers have been dealing with [6].

The transitioning period from regular to remote and online teaching created an urgency for teachers' proficiency in digitalization [7]. Students are skilled in taking part in digital lessons, but teachers' development in that sense turns out to be a lot more difficult [8]. Teachers had to become aware of synchronous and asynchronous learning, their characteristics, the benefits and, most importantly, the necessity to combine both to have quality online education.

To achieve necessary flexibility in teaching and learning in an online environment, detailed planning of all activities that should be implemented is needed. Particular focus must be on selecting the appropriate strategies and digital tools that can be used, considering the limited time for a virtual classroom activity. To have more time for tasks acquiring higher-order thinking skills (problem solving, case analysis, or project development) during virtual classes, teachers should plan different asynchronous activities using the flipped classroom approach [9].

A flipped classroom is a pedagogical approach where direct instruction is transferred from the collective learning space to the interactive individual learning space [10]. It proposes to carry out certain learning processes outside the classroom and use the virtual classroom to enhance the learning process by applying the knowledge and concepts creatively [11]. The potential of this approach lies in the fact that the time invested in explaining the subject through lectures can be designated as work that the students can do at home using video recordings, a narrated presentation, text writings, or self-paced games. Thus, teachers can spend more time promoting interaction in the classroom, guaranteeing higher understanding, and reinforcing the content. Asynchronous activities after the virtual class should be designed to reflect on the learning and to empower students' knowledge and skills. They can refer to different activities, such as additional research, solving nonroutine problems, and students' self-assessment. This approach, gaining in popularity, has seen its value grow in the context of forced social distancing, like the situation we are facing during the pandemic.

Finding a way to keep students' interest and persistence in learning is an important issue that online education is facing, especially during asynchronous activities. It is obvious that this educational change must be tied to the tools and resources students use in their everyday lives. Proper integration of such tools and resources during the learning can increase students’ interest and motivation in the educational process.

Students’ engagement plays a significant role in their achievement within the student-centered learning process [12]. With governments interested in measuring students’ outcomes [13] and findings that student engagement can act as a proxy for quality [14], a clear understanding of student engagement becomes essential. Student engagement is a complex process that brings together diverse research threads contributing to student success explanations [15]. It can lead to the acquisition of long-lasting knowledge and skills necessary for living in this constantly changing world.

One possible way for establishing engaging and interactive learning environments, using students’ energy and enthusiasm while playing games for educational purpose, is the use of game-based learning activities and gamification of different aspects of the educational process.

Educational games increase students' engagement in the learning process. Digital educational games can create new types of interactive learning experiences by combining both game design and instructional design approaches. These interactive learning experiences can increase students' engagement and speed up the process of achieving learning outcomes in the digital learning environment, similar to the way this is achieved in the face-to-face learning environment [16].

According to Sung and Hwang [17], digital games enable problem-solving, decision-making, and strategic planning skills. By using games, new and powerful learning methods in the classroom can be created [18]. By using games, students explore, question, discover, create, and evaluate essential skills that should be acquired. Analysis of engagement in games can provide valuable insights into game mechanisms that can then be applied to games for learning [19].

Gamification, achieved by adding game elements, such as reward and competition during the learning process, provides visible incentives for students' behavior and can increase cognitive load and achievement levels [20]. It can support learning by providing collaboration and self-guided study and increasing motivation, engagement, creativity, and retention [21]. Although gamification and game-based learning are often considered to be synonyms, these 2 concepts are distinct. Game-based learning refers to adopting a game as an educational tool for learning a specific subject in this context. In contrast, gamification is an educational strategy based on game elements in the learning process [22]. Technology-enhanced game-based learning and gamification can increase positive educational results, especially in challenging learning environments [23-25].

The implementation of learning scenarios that use technological resources in and out of distance learning environments face different challenges. It is challenging to match successful games to the curriculum and to use them in the educational process [26]. To achieve valuable integration of games in education, teachers need to connect pedagogical approaches and entertainment [1]. Using interesting games, with features popular among students, for the development of a fun, educational game that leads to the achievement of learning outcomes is 1 possible approach. For example, a mobile-based treasure hunt educational game using augmented reality [27,28] can improve learning experiences by providing active discovery of facts and balancing physical and digital interactions [29]. To encourage the use of games in learning, it is essential to develop an understanding of the tasks, activities, skills, and operations that the games can offer and examine how these match desired learning outcomes while providing a positive learning experience [30]. Most of the games’ usage in the educational process is related to knowledge acquisition and content understanding [31].

An escape room is 1 of the games that are becoming extremely popular among students in the past decade. It is a game where a team of players discovers clues, solves puzzles, and accomplishes tasks in 1 or more rooms to progress and accomplish a specific goal in a limited amount of time [32]. To win the game (“escape”), the players must solve the challenges (puzzles) using hints and clues and developing a strategy [11]. To make this kind of game educational, questions concerning course material can be incorporated within the puzzles. Students then will have to master the material to succeed in the game, which will enhance their learning and increase their interest and engagement in learning [33]. In this way, educational escape room games can improve the skills of students [34]. Escape rooms test the players' problem-solving, lateral thinking, and teamwork skills by providing various puzzles and challenges, making this game perfect for implementation in an educational context [35]. Incorporating educational elements within the challenges leads to achievement of educational goals and increase in students’ motivation and interest in learning [36,37]. Educational escape room games are a new type of learning activity with the promise of enhancing students' learning through highly engaging experiences [25]. Escape rooms have drawn educators' attention due to their ability to foster teamwork and creative thinking in an engaging way for students [33,38]. By adding learning activities to the story in a meaningful way, these escape room games can create memorable learning experiences that cannot be replicated by typical classroom activities [35,39].

Educators have introduced so-called escape room games into their teaching or training practices to solve educational content puzzles [40]. An escape room game contextualizes educational content into a meaningful and inspiring experience based on game-based and collaborative learning [39]. This approach has been used for various subjects [41-43] and various educational process levels [25,44,45].

Developing an escape room in a digital environment suitable for online learning [46,47] is the latest trend in this context. This faces additional challenges because it has to be set up in an online environment, but considering that there are no physical limitations, it can involve a large number of students [48]. The effects of educational digital escape rooms on students' learning performance, learning motivation, and problem-solving ability seem positive [34,45,49].

This paper discusses how we used a design thinking methodology approach for providing a new educational value to a digital escape room game. We started from students’ attitudes toward an already created and played physical escape room game [12], which enabled collaborative learning in the classroom. The idea was to migrate this escape room game to an online environment in order to keep students’ interest and persistence during asynchronous learning. However, due to the new circumstances dictated by the characteristics of an online environment and the short time for its creation, we had to find a new type of engaging experience during the learning process that was not based on collaboration. The digital escape room created was tested by students, and this paper elaborates on whether digital escape room games can increase students' interest and motivation in achieving learning outcomes during remote teaching. Starting from this, the research questions were:

- Can a digital escape room–style educational game be used to achieve student engagement in remote teaching and online learning?
- Can we use the design thinking methodology to create an online escape room–style educational game that is interesting and motivational for students?
- Can we use the design thinking methodology to provide novel educational value to the escape room–style educational game?

The first research question was answered by analyzing students’ answers after using a digital escape room–style educational game during the learning process. The second and third questions concerned the used methodology (design thinking) for developing this kind of a game, so they were answered through the results (outcomes) of each phase of the design thinking process.

The next section explains how we used the design thinking methodology to migrate an escape room–style educational game from a physical to an online environment and the methodology we used to evaluate the achieved level of student engagement. In the Results section, the results obtained from the students' surveys focusing on student engagement and achieved educational value are elaborated. A discussion about these results and the limitations of this research, as well as ideas for future work, are presented in the Discussion section. The last section concludes the paper.

## Methods

### Study Design

We used the design thinking methodology to migrate and transform an escape room–style educational game to an online environment. At the same time, we wanted to check whether a digital escape room game can provide educational value and still be engaging for students, even without teacher supervision and a lack of student collaboration.

**Figure 1:**
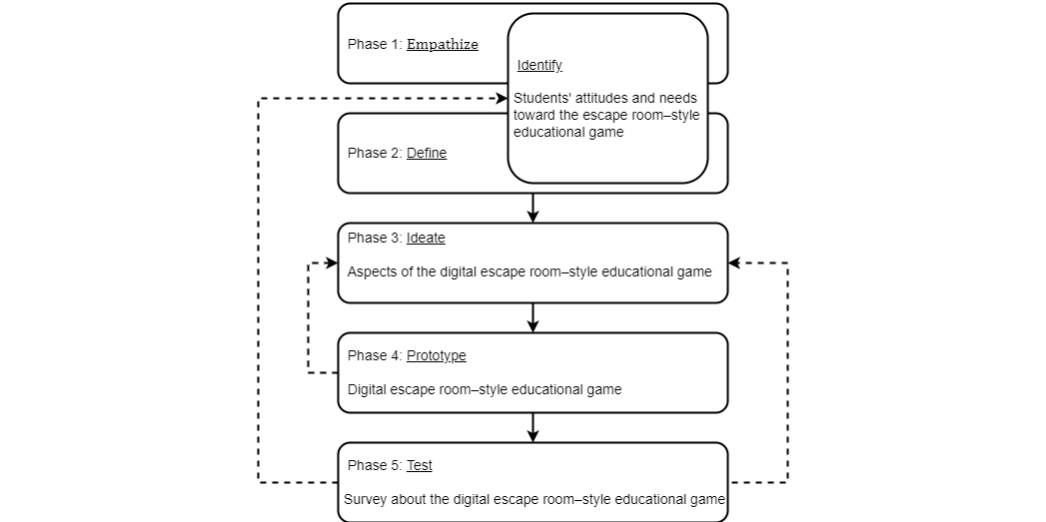
Design thinking methodology used in our study.

The background of design thinking is based on a problem-solving approach [50]—in our case the need to design an online game that will be interesting and will include educational elements at the same time. To achieve this, the digital escape room–style educational game development process was conducted using a modified version of the 5-stage design thinking method proposed by the Hasso-Plattner Institute of Design at Stanford [51-53]. According to this methodological model, the 5 phases of design thinking are *empathize*, *define* (the problem), *ideate*, *prototype*, and *test*. The design thinking methodology applied to our particular case is presented in Figure 1.

Design thinking is a popular methodology that inspires a human-centered approach toward design. For our research, we consolidated the *empathize* and *define* phases into 1 phase, the *identify* phase, which gave us an understanding of the problem from the students' perspective and helped us define the problem in a student-centered manner, starting from their needs and attitudes toward the escape room–style educational game.

Design thinking is a cognitive process from which design concepts emerge [54], and its use suggests that we put emphasis on thinking of design as a process. So, in the next stage, *ideate*, we determined aspects of the digital escape room game, starting from students’ attitudes and the educational elements that should be included.

In the next, *prototype*, phase, we tried to develop a digital escape room–style educational game and test it with students in the testing phase. Students’ feedback from this testing phase will show whether the game corresponds to their needs and whether it is in accordance with the research aims. This feedback will also provide insight into the process and its limitations and can be used for identification of students’ needs as a starting point for another design thinking process in order to improve the quality of the final product—the digital escape room–style educational game.

Although we talk about the design thinking process in terms of sequential steps, it is actually a highly iterative loop. With each phase, we can make new discoveries, which may require revision of the previous stages. The solid lines in Figure 1 represent the chronology of the methodology steps, while the dotted lines represent how different outputs of the design thinking methodology can be used [51].

Each of the phases of the design thinking methodology is explained next.

### Identify Phase: Identification of Students’ Needs and Attitudes Toward the Digital Escape Room–Style Room Educational Game

The first phase of design thinking is to gain an emphatic understanding of the problem we want to solve—how to create a digital escape room–style educational game that will be interesting and educational at the same time and that will engage students in the learning process. To achieve this, we started from students’ attitudes toward escape room–style educational games, and after that, we identified the game aspects that should be adapted for an online environment.

To identify students’ needs and their attitudes toward playing an escape room–style educational game and to test whether this kind of game-based learning can be used for achieving students' engagement during computer science classes, we used already obtained information from the analyses of students’ experiences during the implemented escape room–style educational game in physical surroundings, as described previously [12]. That escape room–style educational game combined computer-based and unplugged puzzles in a computer lab space in order to create a highly engaging activity without compromising its educational value. The puzzles were connected with the course material in general and encouraged research, communication, collaboration, and teamwork. These first findings, presented in detail in our previous work [12], provided evidence that escape room–style educational games constitute a compelling way to achieve student engagement, so transforming this learning experience to an online environment was not a trivial task. The technological restrictions for nonverbal communication and the lack of free movement in a certain physical space created the need to redesign the whole gameplay.

### Ideate Phase: Ideate Aspects of the Digital Escape Room–Style Educational Game

The next phase of the design thinking methodology was to determine necessary aspects of the digital escape room–style educational game. Based on the results of our previous study, we concluded that the collaboration and teamwork that were present in a physical surrounding needed to be replaced with a different aspect of a game that would infuse thrill in a digital escape room game. At the same time, the defined challenges (tasks) needed to have both game-related and novel education-related purposes. To ideate a solution to these nontrivial issues, we started with the results of a survey for applying escape room games in the educational process [12]. Based on those results, we concluded that although the digital version of such game will have no cooperative element, if we manage to keep the thrill and competitiveness, students will still enjoy the game, thus creating a positive and engaging educational experience. To achieve this, we needed to define a new educational value for the game that would replace the collaboration and teamwork required while grasping the concepts of computer science. At the same time, we needed to identify a part of the learning process suitable for individual tasks that could be made more appealing as a game.

### Prototype Phase: Prototyping the Digital Escape Room–Style Educational Game

The prototype phase started by finding a platform where the game would be created and played by students. The platform should enable creating a user-friendly, easy-to-use online game so that students do not have any problems while playing it from a technical aspect. For that purpose, we used a platform that was already used successfully for developing digital escape room games in an educational context [34]. The developed prototype of the digital escape room game was given as an asynchronous activity during online learning. Students got an opportunity to play the game at a time that was most appropriate for them. They would analyze the questions in the puzzles, learn from their mistakes, and reach the end of the game at their own pace.

### Test Phase: Testing the Digital Escape Room–Style Educational Game

Information about students' opinions concerning the digital escape room–style educational game was gathered using an online survey conducted with the students at the end of the activity. The survey was adapted from a similar one on using an escape room–style educational game for teaching programming in a higher education setting [25], and some elements were added from a survey on the implementation of an escape room–style educational game in a physical classroom [12].

The first part of the survey collected demographic information about the participants (gender, age, and school). The second part was designed to measure students’ attitudes toward the game, using a 5-point Likert scale, with answer choices ranging from 1 for strongly disagree to 5 for strongly agree. Information about students' perception about the design of the game, the opportunity to deepen their knowledge, their feelings during play, and their attitude toward this kind of learning activity was obtained. Students got an opportunity to reflect on the game and answer what they like the most, what they did not like, and what can be changed to improve the game through open-ended questions at the end of the survey. In this way, we established a possibility for students to provide inputs for further development of the digital escape room–style educational game (dotted line from Figure 1) by providing constructive feedback about their learning experience.

### Ethical Considerations

The surveys conducted with students are part of everyday practices in schools in order to measure students’ attitudes toward some new teaching approach, and they are completed anonymously by students.

## Results

### Main Goal

The main goal of this work was to evaluate students' perceptions of the implemented digital escape room–style educational game and to determine whether it can be used to achieve student engagement in learning computer science. This was done as part of the process of migration of a traditional collaborative puzzle-based escape room game played in the computer lab to an online environment using the design thinking methodology. In the following sections, the outcomes of each phase of the design thinking process are elaborated. The results in the first, *identify* phase, were obtained from previous research [12] concerning using a physical escape room in education, and it was our starting point for this new research topic, as described in the following phases of the design thinking process.

### Identify Phase: Identification of Students’ Needs and Attitudes Toward the Escape Room–Style Educational Game

The first stage of design thinking was to understand the problem we wanted to solve—how to create a digital game that would be interesting and educational from the students' point of view. We started by identifying popular escape room games among students as part of our previous work. By using a survey, students' attitudes toward using traditional escape room games in education and the elements of the developed escape room–style educational game that students liked the most were identified [12]. The survey results showed that students had a positive overall opinion about the escape room–style educational game and thought it was a fun experience. It was a good starting point for planning the future use of escape room–style games in the educational context, as we wanted to start from students' attitudes toward the game.

Most of the students were competitive during participation in the game, and they were impatient to open the next puzzle, which increased their interest, motivation, and persistence during the game. This competitiveness led to students' active engagement during the game, impatiently trying to be the first to finish the game. These findings provide evidence that escape room–style educational games constitute a compelling way to increase student engagement.

Regarding learning effectiveness, students stated that the escape room game helped them improve their knowledge of computer science and that they prefer the escape room game over other educational methods in computer science classes and think that they can learn more with the game than during regular classes. These were essential findings showing that educational elements can be added in escape room–style games, which can lead to the achievement of needed educational outcomes in a fun and exciting way.

These results were consistent with previous studies, which found that escape room–style educational games can improve students' knowledge of a specific topic [33,37]. However, research shows that escape room–style educational games have been little used, in both compulsory and higher education [55], and that there is not much research concerning the use of the escape room–style educational games in computer science in primary education.

Open-ended questions led to a conclusion that students had enormous interest and motivation in participating in the escape room–style educational game, and most students gave positive comments that they thoroughly enjoyed the game experience and would like more similar future activities. These findings and our previous work [12] gave us a solid base for the next phase of design thinking (*ideate*) to determine aspects of the digital escape room–style educational game.

### Ideate Phase: Ideating Aspects of the Digital Escape Room–Style Educational Game

After understanding students' attitudes and analyzing and synthesizing the feedback provided by students, which enabled approaching a problem from the students' perspective, the next phase in the design thinking process, an idea for creating a digital online game, emerged.

Starting from students' positive attitudes toward using escape room games in education and students' competitiveness while playing the games, we created a digital escape room–style game that would raise students' individual critical-thinking and problem-solving skills, enabling their active participation in the activities.

We concluded that self-assessment is a great candidate for such a process. Self-assessment is an individual learning process, which is important from an educational point of view since it points out the parts of materials that needs more attention by students. In contrast, to serve its purpose, students need to be engaged in the self-assessment process.

The chosen topic to lay behind the story of the digital escape room–style game was connected to algorithmic thinking because the topic is difficult to understand in a traditional teaching environment and should be learned in a fun and interactive way, where students can progress at their own pace according to their previous knowledge. So, learning outcomes connected to algorithmic and computational thinking were planned to be assessed by this digital escape room–style educational game. The educational elements that were supposed to be implemented mostly acquire higher-order thinking skills (creativity, algorithmic thinking, cooperation, critical thinking, and problem solving) [56]. Implementation of these outcomes in coding gives students an opportunity chance to solve problems and to experiment creatively [57]. Students understand that they can learn from their mistakes, which increases their confidence in a fun and exciting way. Developing these skills makes the topic difficult to teach and learn in a traditional classroom and requires more time for learning and enabling students to progress at their own pace. According to this, creating a simple digital escape room–style game where students can solve different questions connected to algorithmic thinking and get an opportunity to self-assess their knowledge and learn from their mistakes was the next step that was carried out in the proposed methodology.

### Prototype Phase: Prototyping the Digital Escape Room–Style Educational Game

**Figure 2:**
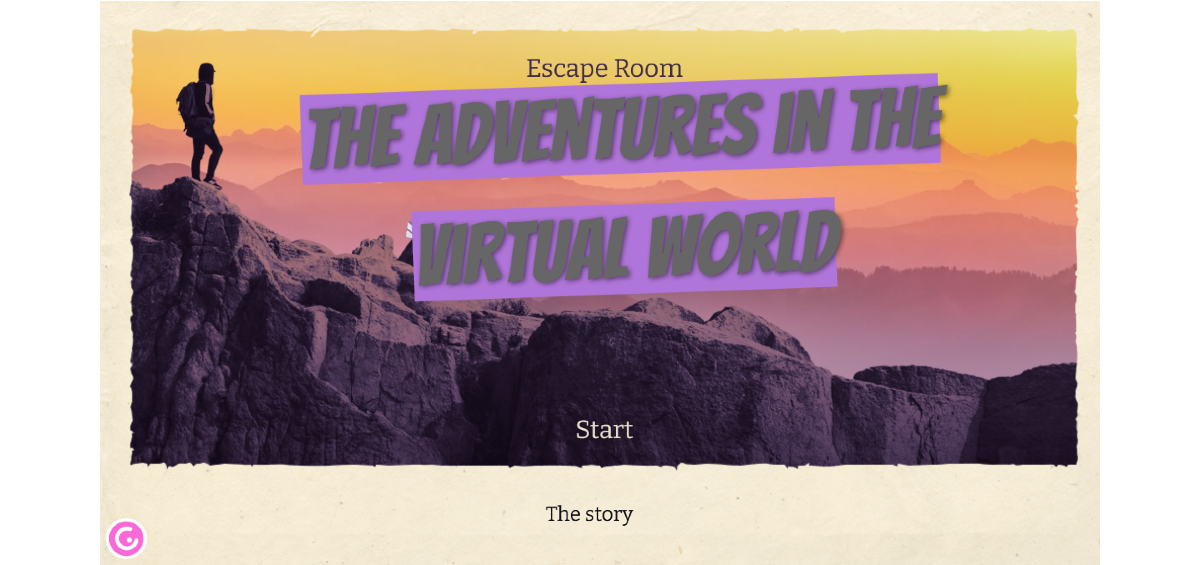
Start page of a digital escape room–style educational game.

A platform that enables creating interactive animated content, Genially [58], was used to create a digital escape room–style educational game. Genially is a platform for creating visually appealing, engaging, interactive digital contents, games, breakout rooms, quizzes, and portfolios. Students could access the game just by following the link given by the teacher, and they could immediately start playing the game. The start page of the created digital escape room–style game is shown in Figure 2.

**Figure 3:**
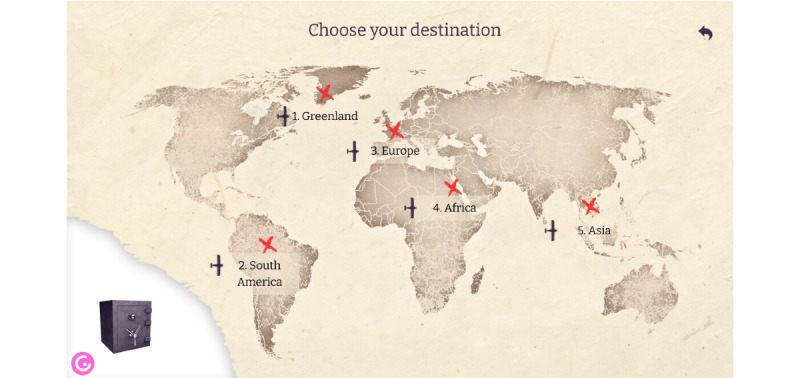
Story behind the puzzles.

Students read the story behind the escape room and start the game. The overall theme of the created escape room is finding the secret to obtaining a higher grade in computer science on a given topic, which is hidden in a coffin. The coffin can be opened using a 5-digit number; each number represents a solution to a different puzzle. The sequence of numbers is important and determines the order of solving the puzzles, so the escape room consists of 5 puzzles hidden in 5 different places, which should be solved. Students virtually travel the world from place to place (Figure 3) and solve puzzles to discover all 5 digits.

**Figure 4:**
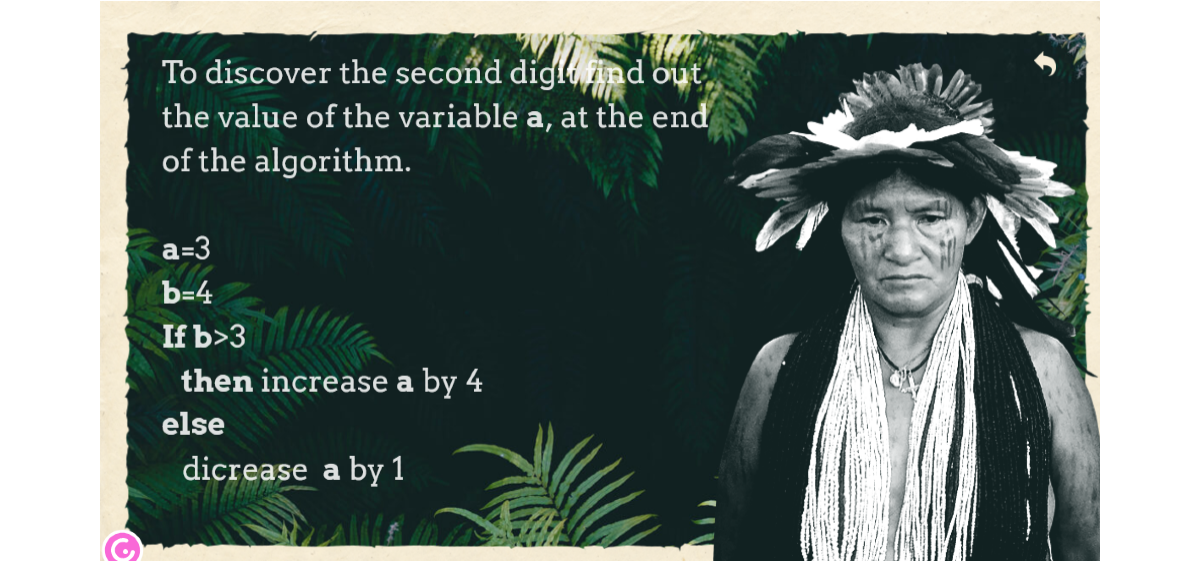
Example of a game’s puzzle.

Identification of the educational elements to be incorporated in the game was conducted. Each puzzle is a question connected to a given algorithm (Figure 4). Puzzles were created carefully, increasing their complexity while going through the game. The first puzzle is the simplest, and each subsequent one uses elements from the previous algorithms. Students analyze each question, pass through the algorithm, and obtain the result—a digit. Finally, students get a 5-digit number used to open а coffin, which is “escape” from the room and end of the game.

This activity was in asynchronous form after the virtual classes. Students implemented the gathered knowledge and skills concerning the topic in concrete situations and got an opportunity to self-assess their knowledge. When playing the digital escape room–style educational game, there was no time restriction. Students passed through the activity at their own pace, at the time that they choose. The questions in the game are time-consuming since they are connected with the development of higher-order thinking skills, which needs time, so there should be no time restriction. However, time was measured, and students knew that they must keep in mind the time necessary to finish the game. The teacher can monitor the ratio of solved tasks in a certain time. This game design was in line with both initial escape room–style educational games and the findings of the educational room student survey (dotted lines in Figure 1).

### Testing Phase: Testing the Digital Escape Room–Style Educational Game

Students answered questions concerning their attitudes about using the digital escape room–style game in an educational context after finishing the game. In total, 117 students from sixth and seventh grades completed the survey to gather information about the achieved student engagement (final phase of the design thinking process), and the results are presented in Table 1. For each question, the mean and SD are presented, showing the dispersion of students' answers. Of the 117 students who played the game, 66 (56.4%) were male and 51 (43.6%) were female. There was no significant difference between the number of students in different classes.

**Table 1:** Results of students' attitudes concerning using the digital escape room–style game in an educational context.

Question	Mean (SD)
It was easy to follow the instructions of the game.	4.44 (0.86)
The puzzles in the game were not difficult to solve.	4.10 (1.17)
The escape room was interesting for me.	5.00 (0)
I was impatient to open the next puzzle and reach the end.	4.41 (1.12)
I learned something new with the game.	4.26 (1.18)
I liked the activity.	4.74 (0.68)
Learning with this kind of activity is more interesting.	4.74 (0.69)
I would like to participate in this kind of activity again.	4.75 (0.67)
The game was complicated.	1.68 (1.19)
I was encouraged to think by the puzzles.	4.64 (0.76)

The results showed that students did not have any problems while playing the online game; they stated that it was easy to follow the game's instructions (mean 4.44, SD 0.86). However, some students faced problems while solving the puzzles, although most of them stated that the puzzles in the game were not difficult to solve (mean 4.10, SD 1.17).

The number of students that found the game puzzles difficult was bigger in digital than in physical surroundings compared to the results of previous research [12]. This was expected because the aim of the puzzles in the digital escape room was not just to engage students but also to involve them in self-assessment, and students had to demonstrate certain knowledge and skills related to the topic to solve the puzzles. Furthermore, the puzzles in the digital escape room require higher-order thinking skills, not just a repetition of facts, because they are connected to 1 of the most difficult themes for students—algorithmic thinking. This requires computational thinking skills, and that is why they are difficult for some of the students. In contrast, the puzzles in the escape room game in physical surroundings are more focused on establishing collaboration and are easier to solve. The SD values showed differences in students' answers, most probably depending on their acquired knowledge about the topic. The results of game complexity (mean 1.68, SD 1.19) are in line with this finding as well.

Interestingly, students were encouraged to think by the puzzles, and they were motivated to think and engage in solving the puzzles (mean 4.64, SD 0.76). So, we could conclude that although some of the students found the puzzles challenging to solve, they were encouraged to think, by which their higher-order thinking skills were developed. Furthermore, they were impatient to open the next puzzle in the escape room game and reach the end (mean 4.41, SD 1.12). The SD values presented a difference in the students' answers and showed that students had different opinions despite a high average value. Once again, this might be the result of the different levels of students' knowledge. Students who did not have problems solving the puzzles were impatient to finish the game sooner, but for students who did not understand the questions, it was hard, and they spent more time on some of the puzzles. After analyzing students’ answers, we found that of 117 students who completed the survey, 90 (76.9%) stated that they did not have problems solving the puzzles and just 16 (13.7%) had some problems in understanding the puzzles’ questions.

Despite the differences in students’ answers about game complexity and puzzles' difficulty, most students liked the activity (mean 4.74, SD 0.68). They enjoyed the game, and they would like to participate in this kind of activity again (mean 4.75, SD 0.67). All 117 (100%) students found the escape room–style game interesting for playing and learning. Thus, we concluded that implementing a digital escape room–style game during online teaching raises students' interest and motivation.

The students' response concerning the educational element of the digital escape room–style game was that they learned something new (mean 4.26, SD 1.18), once again indicating a difference among the students according to their previous experience in the topic and gathered knowledge and skills. Most students found learning with this kind of activity more attractive than other learning forms (mean 4.74, SD 0.69), confirming that interactive learning activities with gaming elements attract students' attention and keep their engagement during the activity.

## Discussion

### Principal Findings

The process of migration of the digital escape room-style game presented in this paper elaborates that the lack of face-to-face collaboration used in knowledge acquisition can be replaced by using an escape room game in different parts of the learning process, for example, self-assessment. In this way, we managed to maintain a high level of student engagement during asynchronous activities, the feature of escape room games that seems to be related to an increase in students’ skills [34].The initial results from the survey related to the use of the digital escape room–style game, as an asynchronous activity, showed that students found the game easy to play, exciting, and engaging and that they would like similar such activities that will last longer and have more challenging puzzles. A few students had some problems while solving the puzzles, but they also found the activity enjoyable.

Students’ answers to the open-ended questions showed that they found the game engaging. Students liked the whole game, moving through the puzzles, logical thinking, and finding the final solution. They mentioned that they learned something new and enjoyed while doing it, which was a completely new experience. They liked that this assessment activity was more like a game, and they had fun while doing it. Learning while playing was stated as 1 of the things that students liked the most. Some of the students liked the escape room's story, how the questions were made and given in the puzzles. They liked the mystery, excitement, and unpredictability and reaching the end of the game. They stated that they liked the overall experience. When students were asked what they liked the least in the online game, they stated that there were a small number of questions and so the game ended quickly. Some of them mentioned that some of the questions were difficult to solve, but they enjoyed the overall experience. These findings are in line with the values of the SD for questions concerning the puzzles' difficulty.

Students said they would like to have more questions of this type for future activities, more puzzles, and even more questions with different complexity levels. Most of the students said they would enjoy more extended challenges. This shows that students enjoyed the game. They would like similar activities soon and even have ideas on how this game can be made more complex and challenging.

All these findings lead to the conclusion that students find our game inspiring and motivational, which confirms that digital escape room–style educational games can be used to achieve student engagement in remote teaching and online learning (our first research question).

Using a design thinking approach, we changed our research focus from being problem oriented to being solution focused and action oriented toward creating a digital escape room–style educational game that would link pedagogical approaches and entertainment elements, starting from students' needs and attitudes toward this topic. During the process, a well-received digital educational game was designed, which was confirmed by students. All students found the game interesting. The analysis of open-ended questions showed that students liked the game, they found it interesting, and they liked solving the puzzles and moving through the game. This is a confirmation that using a design thinking approach, engaging digital educational games that raise students’ interest and motivation can be developed (our second research question).

This study elaborates on how design thinking can be used not only to solve a problem (migration of a game from a traditional to an online learning environment), while preserving its most appreciated elements (engagement) but also, by using the same approach, to reach a new educational value, in our case student self-assessment for the learning process (our third research question).

### Limitations and Future Work

The main drawback of the migration of an escape room game to an online environment is that due to the COVID 19 pandemic circumstances, students have different types of internet infrastructure at their disposal, so the collaborative value of the escape room game could not be implemented in the short period that was available for the migration of the escape room game. There is evidence in the literature [45] of successful deployment of collaboration within a digital escape room–style educational game related to software engineering in higher education, but it should be considering that to create the escape room–style educational game, several highly interactive ad hoc web apps had to be developed from scratch by the course teachers. This was confirmed by López-Pernas et al [43] in a comparative study of the results obtained by face-to-face collaboration and a digital escape room–style educational game used to learn programming. There, the students who participated in the remote escape room–style educational game, due to a lack of a common device, requested and obtained nearly twice as many hints as those who participated in the face-to-face collaboration.

Simple transfer of the physical escape room–style educational game to an online environment using Google Forms and videoconferencing tools was presented by Ang et al [59]. Although students enjoyed the digital escape room game, they felt that a real-life escape room would be more fun and interactive.

The possibility for a large class to use an escape room game simultaneously in or outside the usual classroom was presented by Monnot et al [48]. The authors discussed how the escape room game can also be used as a course support or as preparation for a course, such as work at home, but did not present any evidence of the benefits of this idea.

The main limitation of our study is that only 1 instrument, a student survey, was used to evaluate the effects of the digital escape room–style educational game in the learning process. Although student engagement can be determined using surveys, and the literature confirms that the use of educational games can lead to improved student skills [34], further research related to the achievements of educational outcomes can be used to confirm the stated engagement.

Additional limitations are related to the students’ age and cultural background. Further research is needed to check whether the same engagement based on digital games can be established with younger age groups or with students from different cultural backgrounds.

Our future research will focus on the ways in which escape room games support collaboration, trust, and reflection, thus fostering a dynamic and flexible learning environment [60]. For this purpose, we will investigate the influence of new technologies in establishing such educational games and education in general, such as ubiquitous computing, pervasive computing, virtuality continuum, ambient intelligence, and wearable computing [61]. We will investigate how using escape room games for student assessment can influence students' educational achievements, considering both negative [62] and positive [63] aspects of this approach.

### Conclusion

Student engagement during the learning process is an important element that influences students' learning and determines achieved knowledge and gathered skills. Integration of games that combine entertainment and educational elements during the learning has been recognized as a vital factor for increasing students' interest and motivation during learning. Our previous work confirmed that escape room–style educational games could be used as an engagement activity for students in the classroom environment. Since we have transferred education online due to the pandemic, it was necessary to see whether we can migrate this type of game to an online environment. It was not a trivial process since the main educational benefits of such a game, student engagement through collaboration and teamwork, needed to be transformed and changed into something new in a short period.

In this paper, we presented the process of migration of an escape room–style educational game to an online environment by applying the design thinking methodology. We started with the students' opinion about the use of an escape room–style educational game for learning. Using their opinion, we created a simple digital escape room–style educational game where students got an opportunity to self-assess their knowledge in computer science at their own pace. After playing the game, students' opinions were collected and used to evaluate the process of migration of the escape room–style educational game to an online environment. By using a design thinking approach, we managed to migrate an educational game that links pedagogical approaches and entertainment elements to an online environment and added additional value to it (student self-assessment). The results clearly show that the digital escape room–style educational game was accepted as highly interesting and can be used in remote teaching. The student survey results confirmed that this approach is beneficial for both students and educators. These findings provide a base for our future work. We will explore how digital escape room games can create collaboration and what the influence of new technologies is in establishing this type of educational games. We will focus on escape room games for student assessment and their positive and negative influence on students' educational achievements. We believe that this kind of research can provide a completely new view of pedagogical approaches applicable in distance learning environments.
